# Masculine Norms and Infectious Disease: The Case of COVID-19

**DOI:** 10.1017/S1743923X20000380

**Published:** 2020-07-07

**Authors:** Tyler T. Reny

**Affiliations:** Washington University in St. Louis

**Keywords:** COVID-19, infectious disease, sexism, masculinity, political behavior, public health

## Abstract

During the novel coronavirus pandemic, early data suggested that men were slightly more likely to contract COVID-19 than women, less likely to seek medical attention for the disease, and far more likely to die as a result of COVID-19. While several studies have explored this gender gap, none has attempted to isolate the psychological processes underpinning this phenomenon. In this research note, I suggest that sexism partly explains these differences. Using data from a large (*N* = 100,689) survey of American adults conducted between March and June 2020 by the Democracy Fund and the University of California, Los Angeles (Nationscape), I find that sexist beliefs, a component of masculine norms, are consistently the strongest predictor of coronavirus-related emotions, behaviors, policy attitudes, and ultimately contracting COVID-19. This study highlights how gender ideology can impact health and impede government public health efforts.

In late May 2020, when the United States had just surpassed 500,000 confirmed COVID-19 cases and was nearing 100,000 deaths, President Donald Trump refused to don a mask during a visit to a mask factory in Michigan. The president claimed that he did not want to give the press the “pleasure of seeing it.” He later mocked Democratic presidential candidate Joe Biden for wearing a mask. Trump's refusal to “look weak” highlights how attitudes about masculinity could impede efforts by public health officials to stem the spread of infectious disease.

The vast majority of political science research on the coronavirus pandemic thus far has examined the role of partisan identity. Several researchers have argued that partisanship is among the most significant and consistent factors differentiating health behaviors and policy attitudes (Allcott et al. 2020; Gadarian et al. 2020; Pickup, Stecula, and van der Linden 2020). Focus on elites and partisan identity, however, ignores the role that commitments to masculine norms, which cut across predispositions and demographics, can play in shaping health behaviors and preferences.

Building on research in public health and political science, I argue that masculine norms play understudied but crucial roles in shaping health behaviors and preferences during the coronavirus pandemic, particularly at a time when messages from elites reinforce the link between these attitudes and health behaviors.

Using a large national survey of more than 100,000 respondents fielded between March and June 2020 by the Democracy Fund and the University of California, Los Angeles (UCLA), I explore the correlates of pandemic-related outcomes. I find that sexism, a component of masculine belief systems, predicts lower levels of concern about the coronavirus, lower levels of engagement in precautionary behaviors, lower levels of support for state and local pandemic policies, and ultimately higher levels of COVID-19 sickness. Sexism is among the strongest correlates of these outcomes, stronger even than partisanship, ideology, gender, and education.

## GENDER AND PUBLIC HEALTH BEHAVIORS

Public health researchers have long explored how gender shapes public health outcomes. Men have higher levels of negative emotional states, are less likely to seek out physical or mental health services, and are more likely to engage in risky behaviors and exhibit poorer physical and mental health outcomes (see Courtenay 2000 for an overview).

Underlying these gaps is a social construction of gender roles, behaviors, and performance (Kimmel 1995). In many countries, the socially dominant conception of traditional gender norms idealizes men as independent, self-reliant, and tough and women as protective and weak (Martin 1995). Belief in these gender norms is reflected in destructive health behaviors such as denial of weakness and vulnerability, dismissal of the need for help, hiding of disability or illness to avoid seeming feminine or weak (Charmaz 1995; Courtenay 2000; Levant et al. 2009; Yousaf, Popat, and Hunter 2015), and support for a variety of related political outcomes (McDermott 2016).^1^

More importantly these health behaviors also serve to sustain and reproduce structures of power (Pyke 1996). As with President Trump's refusal to wear a face mask in public and his criticism of Joe Biden for doing so, health behaviors can demonstrate masculine ideals that serve to reinforce the systematic subordination of women or “weak” men and preserve hierarchies of authority (McDermott 2016).

Importantly, because gendered ideology is socialized, it can be adopted by women and reflected in women's health behaviors as well. Sloan, Conner, and Gough (2015) find that aspects of masculinity predict worse health behaviors for both men and women. Therefore, this study hypothesizes that gendered ideology, as measured by sexist beliefs that reaffirm men's position in social hierarchies, will be predictive of lower levels of concern about the coronavirus pandemic, less engagement in precautionary behavior, less support for pandemic policies, and finally higher levels of illness regardless of one's gender, race, or partisan identity.

## DATA AND METHODS

To test these expectations, this study uses national repeated cross-sectional survey data from Nationscape, an ongoing weekly online survey (*n* ~ 6,250/week) conducted by the Democracy Fund and UCLA (Tausanovtich and Vavreck 2020) that is weighted to be representative of the U.S. national population (see Tausanovitch et al. 2019). I include all waves that asked questions about COVID-19 (March 19, 2020–June 4, 2020), rendering a total sample of *N* = 100,689. This survey contains multiple questions that tap into COVID-19 concern, behaviors, policy preferences, and self-reported sickness.^2^

The independent variable in my analyses is an additive index measuring sexist attitudes. The scale comprises four questions tapping into several components of sexism, including a belief in the biological superiority of men over women—old-fashioned sexism (Swim et al. 1995)—as well as beliefs that gender hierarchies should be maintained (the measure has been rescaled to range between 0 and 1; M = 0.32, SD = 0.18).

The dependent variables are items measuring (1) coronavirus concern (4 = very concerned), (2) self-reported precautionary behaviors (1 = yes), (3) attitudes toward pandemic-related policies (4 = strongly agree), and (4) contracting COVID-19 (1 = yes).

I regress each outcome on the sexism index and control for a host of standard confounders, including education (1 = college), partisanship (7 = strong Republican), ideology (5 = very conservative), race (white = 1), old-fashioned racism (4 = strongly agree), gender (1 = female), age, political interest (4 = most of the time), household income, population density (logged zip code population), and employment (1 = unemployed). All regressions use survey weights and include fixed effects for survey wave. For additional information on question wording, descriptives, and scales see Appendix B in the supplementary materials.

## ANALYSIS

**Figure 1.**
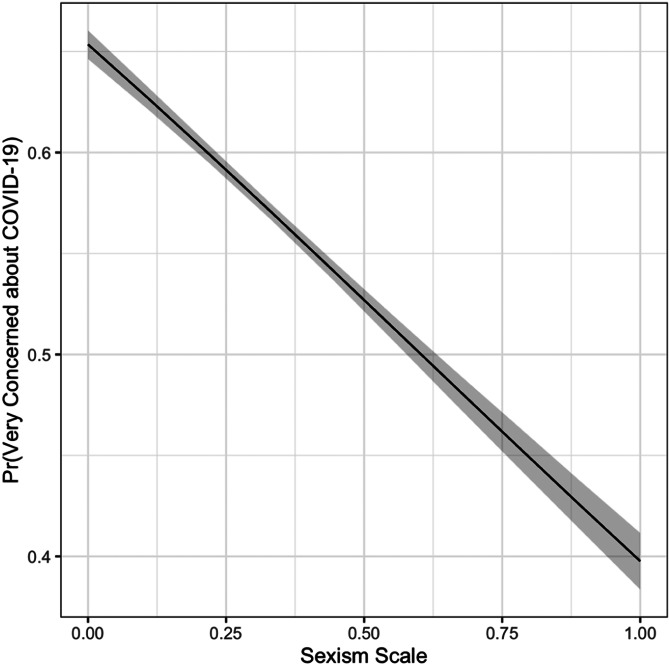
Sexism and concern. Simulated probability of being “very concerned” about coronavirus. 95% confidence intervals. Full regression table in Appendix C.

I begin by using an ordered probit regression to examine whether those high in sexism report being more concerned about the coronavirus than those lower in sexism, all else being equal. In Figure 1, I plot the predicted probability of reporting being “very concerned” moving from lowest to highest values of sexism and holding all other variables at their means. Those high in sexism are 26 percentage points less likely to report being “very concerned” than those low in sexism. This association is stronger than that of partisanship, ideology, race, education, or gender, and it is only matched by the magnitude of the relationship between age and concern.

**Figure 2.**
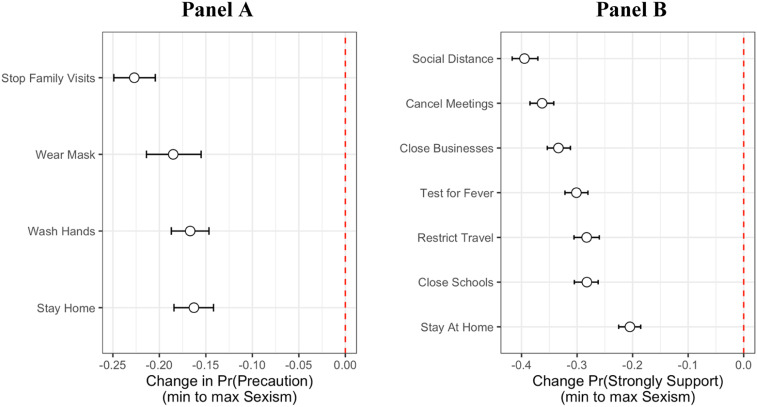
Sexism, precautions, and policy attitudes. Change in probability of (a) engaging in precautionary behavior or (b) strongly supporting state and local policies. 95% confidence intervals. Full regression table in Appendix C.

Next, we might assume that this lack of concern among those high in sexism would be reflected in both lower levels of precautionary behavior and lower support for policies targeted at impeding the spread of the coronavirus. In Figure 2, Panel A, I present the change in the probability of engaging in four behaviors: (1) stopping visiting family and friends, (2) wearing a mask while outdoors, (3) washing hands more than usual, and (4) self-quarantining at home, moving the sexism scale from its observed minimum to maximum holding all other variables at their means. Consistent with expectations, those highest in sexism were between 17 and 23 percentage points less likely to engage in these precautionary behaviors. The same holds for policy support. In Panel B, I show that those highest in sexism are between 21 and 39 percentage points less likely to strongly support these state and local policies than those lowest in sexism, all else being equal.

**Figure 3.**
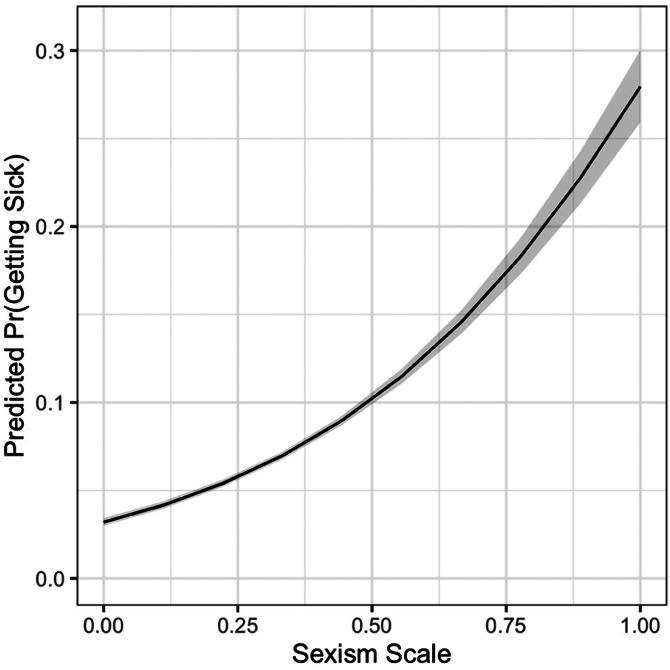
Sexism and contracting COVID-19. Simulated probability of reporting having contracted COVID-19. 95% confidence intervals. Full regression table in Appendix C.

Finally, if those highest in sexism are less likely to be concerned about the coronavirus and less likely to take precautions, it is likely that they would also be more likely to contract COVID-19. In Figure 3, I plot the predicted probability that a respondent indicates that they have or may have gotten sick with the coronavirus. On average, 3.2% of those lowest on the sexism scale report having gotten sick, while 28% of those highest on the sexism scale say the same.^3^

## CONCLUSION AND DISCUSSION

When it comes to public health directives from government officials during an infectious disease pandemic, it is clear that predispositions such as partisanship could shape individual responses. Few have yet focused on the role that gender ideology can play in shaping behavior, particularly at a time when the U.S. president openly modeled these norms.

In this research note, I use a large national survey of American adults to estimate the relationship between sexist attitudes and emotional, behavioral, and attitudinal responses to the coronavirus pandemic. I find that sexist individuals are less likely to be worried about the coronavirus, less likely to engage in behaviors to protect themselves and others, less likely to support state and local government policies that aim to stem the spread of the disease, and finally, are more likely to get sick themselves. Together, these findings suggest that messaging around public health measures need to overcome barriers around the perceived “masculinity” of behaviors, as Representative Nancy Pelosi modeled during an interview with CNBC, when she remarked that “real men wear masks.”^4^

While this study finds that sexist attitudes are strongly correlated with coronavirus behaviors and attitudes, it does little to dig into the proposed mechanism underlying this relationship—specifically gendered personality or masculine norm adherence. Future work could use developed measures such as the Bem Sex Role Inventory to explore these relationships. Further, future work could leverage survey experiments to assess the role that elite messages or behaviors play in shaping perceptions of health behaviors as masculine or feminine and how those perceptions spill over into mass attitudes and behaviors.

## Footnotes

1. For more on the role of gendered personalities and sexism in American politics, see Appendix A in the supplementary materials.

2. For more information on the Nationscape survey, see Appendix B in the supplementary materials.

3. I show in Appendix C that these findings hold with male/female, white/nonwhite, and Democratic/Republican split samples as well as controlling for approval of President Trump.

4. Jacob Pramuk, “‘Real Men Wear Masks’: Nancy Pelosi Urges Caution as the Economy Reopens,” CNBC, June 4, 2020, https://www.cnbc.com/2020/06/04/coronavirus-pelosi-urges-americans-to-wear-masks-pushes-for-relief-bill.html (accessed July 30, 2020).

## Supplementary material

For supplementary material accompanying this paper visit https://doi.org/10.1017/S1743923X20000380. click here to view supplementary material
