# Supplementary material for: Masculine Norms and Infectious Disease: The Case of COVID-19
Source: Politics & Gender. 2020 Jul 7:1–8. doi: 10.1017/S1743923X20000380 (PMC7477467; doi:10.1017/S1743923X20000380)
Supplement: Supplementary file 1 [file S1743923X20000380sup001.pdf]

Online Appendix for "Masculine Norms and Infectious Disease: The Case of COVID-19"

A Sexism in American Politics

Sexism is uniquely important during the coronavirus pandemic for another reason: President Donald Trump. Scholars have shown that sexism has been playing an increasingly central role in American politics. Sexism conditioned reactions to Donald Trump's campaign attacks on Hillary Clinton (Cassese and Holman 2019; Schaffner et al. 2018), was correlated with support for Trump over Clinton (Valentino et al. 2018; Schaffner et al. 2018; Sides et al. 2018) among both white men and women (Cassese and Barnes 2019; Frasure-Yokley 2018; Bracic et al. 2019), and has continued to exert influence in other down ballot elections like the 2018 U.S. midterms (Schaffner 2020). Similarly, gendered personalities are correlated with a variety of political outcomes like identification with and support for the Republican Party, levels of political engagement and interest in politics, and stronger beliefs in traditional gender roles in political and social life (McDermott 2016). It is likely, then, that Trump's brash display of machismo, by going without wearing a mask, taps into an already heightened association among politically aware individuals between masculinity norms, Trump, and the Republican Party.

B Questions, Descriptive Statistics, and Scale Construction

B.1 Nationscape Data

Nationscape is a large, weekly online survey conducted by Lucid for the Democracy Fund and researchers at UCLA that was designed to collect weekly snapshots of the American electorate throughout the 2019-2020 primary and general elections (Tausanovitch and Vavreck 2020). This cross-section survey is in the field every day of the week and includes weekly collections of N$\sim$6,250 responses. While the sample is opt-in, a representativeness assessment of the data finds that the samples are comparable to those collected by well-known pollsters like Pew and YouGov (Tausanovitch et al. 2019). More information on the survey can be found at https://www.voterstudygroup.org/nationscape.

B.2 IV: Sexism Scale

Respondents indicated whether they agreed or disagreed (5-pt Likert) with the following statements. The first two are part of an established old-fashioned sexism battery (Swim et al. 1995) and the last two, which tap into perceptions of attitudes toward gendered social hierarchies, were created by researchers at Democracy Fund Voter Study Group for their panel study.

- "I would be more comfortable having a man as a boss than a woman" (reversed, mean=2.82)

- “Women are just as capable of thinking logically as men” (mean=1.66)
- “Increased opportunities for women have significantly improved the quality of life in the United States” (mean=2.03)
- “Women who complain about harassment often cause more problems than they solve” (reversed, mean=2.70)

Responses for each range from 1=strongly disagree to 5=strongly disagree. While these measures do not directly measure adherence to masculine norms, sexism is a central component of the masculinity belief system and is highly correlated with conforming to masculine norms (Smiler 2006). Questions were added together into a sexism scale (α=0.56) that was re-scaled to range between 0 and 1 (mean=0.32, sd=0.18).

B.3 DV: Behaviors, Attitudes, and Sickness

The dependent variables are a series of items measuring: (1) concern about coronavirus; (2) self-reported precautionary behaviors; (3) attitudes toward pandemic-related state and local policies; and (4) whether the respondent had or has contracted COVID-19. Full question wording below:

Concern

- “How concerned are you about coronavirus here in the United States?” (1=Very concerned; 2=Somewhat concerned; 3=Not very concerned; 4=Not at all concerned; mean=1.57)

Precautionary Behavior

Have you done any of the following things in response to the spread of coronavirus?

- *Wash Hands* “Washed your hands more often than you typically do?” (1=Yes; 0=No; mean=0.92)
- *Wear Mask* “Worn a mask when going out in public?” (1=Yes; 0=No; mean=0.84)
- *Stop Family Visits* “Stopped visiting family or friends?” (1=Yes; 0=No; mean=0.81)
- *Stay Home* “Not left my home for a prolonged period of time?” (1=Yes; 0=No; mean=0.78)

State and Local Policies

“As you may know, some state and local governments have taken certain actions in response to the coronavirus and are considering other actions. Do you support or oppose the following actions?”

- *Cancel Meetings* “Cancel all meetings or gatherings of more than 10 people, like sports events, concerts, conferences, etc” (1=Strongly support; 2=Somewhat support; 3=Somewhat oppose; 4=Strongly oppose; 5=Don’t know (recoded as NA); mean=1.65)
- *Close Businesses* “Close certain businesses where larger numbers of people gather, like theaters, bars, restaurants, etc.” (1=Strongly support; 2=Somewhat support; 3=Somewhat oppose; 4=Strongly oppose; 5=Don’t know (recoded as NA); mean=1.67)
- *Close Schools* “Close schools and universities” (1=Strongly support; 2=Somewhat support; 3=Somewhat oppose; 4=Strongly oppose; 5=Don’t know (recoded as NA); mean=1.69)
- *Restrict Travel* “Restrict travel by plane, train, or bus” (1=Strongly support; 2=Somewhat support; 3=Somewhat oppose; 4=Strongly oppose; 5=Don’t know (recoded as NA); mean=1.68)
- *Stay at Home* “Restrict all non-essential travel outside the home” (1=Strongly support; 2=Somewhat support; 3=Somewhat oppose; 4=Strongly oppose; 5=Don’t know (recoded as NA); mean=1.92)
- *Social Distance* “Encourage people to stay in their homes and avoid socializing with others” (1=Strongly support; 2=Somewhat support; 3=Somewhat oppose; 4=Strongly oppose; 5=Don’t know (recoded as NA); mean=1.57)
- *Test for Fever* “Test people for a fever before letting them enter public buildings” (1=Strongly support; 2=Somewhat support; 3=Somewhat oppose; 4=Strongly oppose; 5=Don’t know (recoded as NA); mean=1.67)

Sick with COVID-19

Have any of the following people been sick with coronavirus?

- *Sick* “You” (1=Yes/Maybe; 0=No; mean=0.087)

Control Variables

- *College* (1=Bachelor’s Degree or greater; mean=0.30)
- *Partisanship* (1=Strong Democrat; 2=Weak Democrat; 3=Lean Democrat; 4=Independent; 5=Lean Republican; 6=Weak Republican; 7=Strong Republicans; mean=3.90)
- *Ideology* (1=Very liberal; 2=Liberal; 3=Moderate/Not Sure; 4=Conservative; 5=Very Conservative; mean=3.04)
- *Gender* (1=Female; 0=Other; mean=0.52)
- *Age* (Continuous; mean=47.46)

- *Race* (1=Non-Hispanic White; 0=Other; mean=0.66)
- *Old-Fashioned Racism* (Additive index of two old-fashioned racism questions below scaled between 0 and 1. mean=0.34)
  - "I prefer that my close relatives marry spouses from their same race" (Strongly agree=5; Somewhat agree=4; Neither agree nor disagree=3; Somewhat disagree=2; Strongly disagree=1)
  - "I think it's alright for blacks and whites to date each other" (Strongly agree=1; Somewhat agree=2; Neither agree nor disagree=3; Somewhat disagree=4; Strongly disagree=5)
- *Interest* (1=Most of the time; 2=Some of the time; 3=Only now and then; 4=Hardly at all; mean=1.83)
- *Household Income* (1=Less than $14,999 to 24=$250,000 and above; mean=12.89)
- *Unemployed* (1=Yes; 0=No; mean=0.10)
- *Logged Population* (mean=12.66)

C Regression Tables

Table 1: Concern

	Dependent variable:
	Coronavirus Concern
Sexism	-0.655^{***}
	(0.026)
College	0.097^{***}
	(0.010)
Democrat	0.304^{***}
	(0.012)
Republican	-0.071^{***}
	(0.012)
Conservative (5-pt Ideology)	-0.158^{***}
	(0.005)
Female	0.184^{***}
	(0.008)
Age	0.010^{***}
	(0.0002)
White	-0.197^{***}
	(0.009)
Racism	0.262^{***}
	(0.018)
Political Interest	-0.203^{***}
	(0.005)
Household Income	0.002^{**}
	(0.001)
Unemployed	-0.005
	(0.014)
Logged Population	0.030^{***}
	(0.002)
Wave FEs	Yes
Observations	90,722
Note:	*p<0.1; **p<0.05; ***p<0.01

Ordered probit regression coefficients. Standard errors in parentheses. Two-tailed t-tests. All models use survey weights.

Table 2: Precautions

	Dependent variable:			
	Wash Hands	Stop Family Visits	Stay Home	Wear Mask
	(1)	(2)	(3)	(4)
Sexism	-1.870^{***}	-1.397^{***}	-0.906^{***}	-1.338^{***}
	(0.085)	(0.061)	(0.056)	(0.096)
College	0.152^{***}	0.308^{***}	0.219^{***}	0.285^{***}
	(0.035)	(0.025)	(0.022)	(0.039)
Democrat	0.428^{***}	0.305^{***}	0.208^{***}	0.602^{***}
	(0.039)	(0.029)	(0.027)	(0.044)
Republican	0.142^{***}	-0.013	-0.018	0.127^{***}
	(0.038)	(0.029)	(0.027)	(0.042)
Conservative (5-pt Ideology)	-0.041^{***}	0.010	-0.065^{***}	-0.135^{***}
	(0.015)	(0.011)	(0.010)	(0.017)
Female	0.220^{***}	0.533^{***}	0.381^{***}	0.196^{***}
	(0.028)	(0.020)	(0.018)	(0.031)
Age	0.009^{***}	0.006^{***}	0.001	0.016^{***}
	(0.001)	(0.001)	(0.001)	(0.001)
White	-0.053^{*}	-0.040^{*}	-0.064^{***}	-0.255^{***}
	(0.031)	(0.022)	(0.021)	(0.036)
Racism	0.096	-0.236^{***}	-0.079^{**}	-0.415^{***}
	(0.060)	(0.043)	(0.039)	(0.066)
Political Interest	-0.262^{***}	-0.205^{***}	-0.199^{***}	-0.183^{***}
	(0.015)	(0.011)	(0.010)	(0.017)
Household Income	0.002	0.019^{***}	0.007^{***}	0.015^{***}
	(0.002)	(0.001)	(0.001)	(0.002)
Unemployed	-0.087^{**}	-0.138^{***}	0.131^{***}	-0.334^{***}
	(0.043)	(0.031)	(0.031)	(0.047)
Logged Population	0.097^{***}	0.081^{***}	0.070^{***}	0.193^{***}
	(0.008)	(0.006)	(0.005)	(0.009)
Intercept	1.812^{***}	0.009	0.501^{***}	-0.613^{***}
	(0.143)	(0.101)	(0.094)	(0.157)
Wave FEs	Yes	Yes	Yes	Yes
Observations	77,427	77,254	77,339	37,626
Log Likelihood	−19,226.680	−32,413.390	−37,393.480	−13,858.210
Akaike Inf. Crit.	38,499.360	64,872.770	74,832.960	27,752.410
Note:				*p<0.1; **p<0.05; ***p<0.01

Logistic regression coefficients. Standard errors in parentheses. Two-tailed t-tests. All models use survey weights.

Table 3: Policy Support

	Dependent variable:						
	Cancel Meet	Close Business	Close School	Restrict Travel	Stay At Home	Social Distance	Test for Fever
	(1)	(2)	(3)	(4)	(5)	(6)	(7)
Sexism	−0.945***	−0.862***	−0.723***	−0.726***	−0.533***	−1.040***	−0.776***
	(0.029)	(0.028)	(0.028)	(0.032)	(0.027)	(0.032)	(0.028)
College	0.038***	0.034***	−0.008	−0.021*	0.055***	0.031**	0.020*
	(0.011)	(0.010)	(0.010)	(0.012)	(0.010)	(0.012)	(0.010)
Democrat	0.182***	0.198***	0.139***	0.111***	0.161***	0.206***	0.135***
	(0.014)	(0.014)	(0.014)	(0.016)	(0.013)	(0.016)	(0.014)
Republican	−0.157***	−0.142***	−0.179***	−0.088***	−0.120***	−0.108***	−0.059***
	(0.014)	(0.014)	(0.014)	(0.016)	(0.014)	(0.016)	(0.014)
Conservative (5-pt Ideology)	−0.133***	−0.127***	−0.117***	−0.088***	−0.106***	−0.099***	−0.059***
	(0.005)	(0.005)	(0.005)	(0.005)	(0.005)	(0.006)	(0.005)
Female	0.199***	0.178***	0.161***	0.195***	0.170***	0.203***	0.162***
	(0.009)	(0.009)	(0.009)	(0.010)	(0.009)	(0.010)	(0.009)
Age	0.008***	0.006***	0.006***	0.004***	0.003***	0.006***	0.008***
	(0.0003)	(0.0003)	(0.0003)	(0.0003)	(0.0003)	(0.0003)	(0.0003)
White	−0.100***	−0.089***	−0.089***	−0.040***	−0.205***	−0.071***	−0.136***
	(0.010)	(0.010)	(0.010)	(0.011)	(0.010)	(0.012)	(0.010)
Racism	0.045**	0.097***	0.025	0.101***	0.181***	−0.027	0.089***
	(0.020)	(0.020)	(0.020)	(0.022)	(0.019)	(0.022)	(0.020)
Political Interest	−0.050***	−0.061***	−0.046***	−0.013**	−0.045***	−0.058***	−0.088***
	(0.005)	(0.005)	(0.005)	(0.006)	(0.005)	(0.006)	(0.005)
Household Income	0.002***	0.002**	0.0002	−0.0003	0.002***	0.005***	0.002***
	(0.001)	(0.001)	(0.001)	(0.001)	(0.001)	(0.001)	(0.001)
Unemployed	−0.049***	−0.012	0.019	−0.038**	−0.060***	−0.057***	−0.041***
	(0.015)	(0.015)	(0.015)	(0.017)	(0.015)	(0.017)	(0.015)
Logged Population	0.021***	0.017***	0.008***	−0.002	0.031***	0.013***	0.009***
	(0.003)	(0.003)	(0.003)	(0.003)	(0.003)	(0.003)	(0.003)
Wave FEs	Yes	Yes	Yes	Yes	Yes	Yes	Yes
Observations	71,842	71,817	71,666	56,837	71,532	57,653	70,758
Note:							*p<0.1; **p<0.05; ***p<0.01

Ordered probit regression coefficients. Standard errors in parentheses. Two-tailed t-tests. All models use survey weights.

Table 4: Contracting COVID-19

	Dependent variable:
	You Got Sick
Sexism	2.460***
	(0.076)
College	0.157***
	(0.028)
Democrat	0.074*
	(0.039)
Republican	0.200***
	(0.039)
Conservative (5-pt Ideology)	−0.304***
	(0.012)
Female	−0.142***
	(0.025)
Age	−0.032***
	(0.001)
White	−0.109***
	(0.027)
Racism	0.830***
	(0.055)
Political Interest	−0.249***
	(0.015)
Household Income	0.021***
	(0.002)
Unemployed	0.075*
	(0.041)
Logged Population	0.027***
	(0.007)
Intercept	−1.473***
	(0.127)
Wave FEs	Yes
Observations	93,724
Log Likelihood	−23,373.630
Akaike Inf. Crit.	46,793.260
Note:	*p<0.1; **p<0.05; ***p<0.01

Logistic regression coefficients. Standard errors in parentheses. Two-tailed t-tests. All models use survey weights.

Table 5: Models by Gender

	Dependent variable:							
	Concern		Wash Hands		Cancel Meetings		You Sick	
	ordered probit		*logistic*		*ordered probit*		*logistic*	
	F	M	F	M	F	M	F	M
	(1)	(2)	(3)	(4)	(5)	(6)	(7)	(8)
Sexism	−0.728***	−0.647***	−2.084***	−1.752***	−0.959***	−0.985***	2.226***	2.592***
	(0.037)	(0.036)	(0.126)	(0.117)	(0.042)	(0.040)	(0.114)	(0.104)
College	0.031**	0.158***	0.085*	0.206***	−0.026*	0.103***	−0.042	0.324***
	(0.013)	(0.014)	(0.049)	(0.049)	(0.015)	(0.015)	(0.042)	(0.038)
Democrat	0.224***	0.386***	0.280***	0.564***	0.163***	0.209***	0.020	0.123**
	(0.017)	(0.017)	(0.058)	(0.054)	(0.020)	(0.020)	(0.056)	(0.054)
Republican	−0.186***	0.035**	−0.034	0.284***	−0.176***	−0.135***	0.151***	0.229***
	(0.017)	(0.017)	(0.058)	(0.051)	(0.020)	(0.020)	(0.058)	(0.054)
Conservative (5-pt Ideology)	−0.132***	−0.173***	0.011	−0.073***	−0.136***	−0.128***	−0.259***	−0.315***
	(0.007)	(0.006)	(0.024)	(0.020)	(0.008)	(0.007)	(0.021)	(0.016)
Age	0.012***	0.008***	0.009***	0.008***	0.010***	0.006***	−0.029***	−0.034***
	(0.0004)	(0.0003)	(0.001)	(0.001)	(0.0004)	(0.0004)	(0.001)	(0.001)
White	−0.229***	−0.170***	−0.00003	−0.112**	−0.137***	−0.064***	−0.083**	−0.173***
	(0.013)	(0.014)	(0.045)	(0.044)	(0.015)	(0.015)	(0.040)	(0.037)
Racism	0.169***	0.346***	−0.045	0.199**	−0.033	0.124***	0.413***	1.182***
	(0.025)	(0.026)	(0.088)	(0.083)	(0.028)	(0.028)	(0.082)	(0.076)
Political Interest	−0.208***	−0.196***	−0.266***	−0.262***	−0.034***	−0.067***	−0.205***	−0.287***
	(0.007)	(0.007)	(0.022)	(0.020)	(0.008)	(0.008)	(0.022)	(0.021)
Household Income	−0.001	0.004***	−0.006**	0.008***	0.001	0.003**	0.019***	0.022***
	(0.001)	(0.001)	(0.003)	(0.003)	(0.001)	(0.001)	(0.003)	(0.003)
Unemployed	0.036*	−0.035*	0.062	−0.178***	0.009	−0.093***	0.023	0.148***
	(0.020)	(0.019)	(0.069)	(0.056)	(0.022)	(0.021)	(0.062)	(0.054)
Logged Population	0.021***	0.036***	0.103***	0.089***	0.024***	0.018***	0.033***	0.021**
	(0.003)	(0.004)	(0.012)	(0.011)	(0.004)	(0.004)	(0.011)	(0.010)
Constant			2.164***	1.751***			−1.707***	−1.563***
			(0.211)	(0.192)			(0.186)	(0.170)
Wave FEs	Y	Y	Y	Y	Y	Y	Y	Y
Observations	47,490	43,232	41,679	35,748	37,913	33,930	50,737	42,987
Log Likelihood			−9,209.862	−9,967.498			−10,959.660	−12,328.090
Akaike Inf. Crit.			18,463.720	19,978.990			21,959.310	24,696.180
Note:								*p<0.1; **p<0.05; ***p<0.01

Regression type noted above columns. Standard errors in parentheses. Two-tailed t-tests. All models use survey weights.

Table 6: Models by Race

	Dependent variable:							
	Concern		Wash Hands		Cancel Meetings		You Sick	
	ordered probit		*logistic*		*ordered probit*		*logistic*	
	W	NW	W	NW	W	NW	W	NW
	(1)	(2)	(3)	(4)	(5)	(6)	(7)	(8)
Sexism	−0.548***	−0.829***	−1.476***	−2.564***	−0.773***	−1.247***	2.222***	2.739***
	(0.031)	(0.045)	(0.105)	(0.151)	(0.035)	(0.050)	(0.098)	(0.124)
College	0.075***	0.139***	0.173***	0.122*	0.026**	0.056***	0.222***	−0.020
	(0.011)	(0.018)	(0.041)	(0.064)	(0.012)	(0.020)	(0.034)	(0.050)
Democrat	0.329***	0.265***	0.426***	0.455***	0.263***	0.084***	−0.103**	0.272***
	(0.016)	(0.019)	(0.051)	(0.062)	(0.018)	(0.023)	(0.052)	(0.059)
Republican	−0.061***	−0.073***	0.181***	0.096	−0.127***	−0.152***	0.109**	0.324***
	(0.015)	(0.022)	(0.047)	(0.069)	(0.017)	(0.026)	(0.050)	(0.065)
Conservative (5-pt Ideology)	−0.189***	−0.095***	−0.124***	0.087***	−0.168***	−0.059***	−0.301***	−0.270***
	(0.006)	(0.008)	(0.019)	(0.026)	(0.006)	(0.009)	(0.015)	(0.021)
Age	0.010***	0.009***	0.011***	0.004**	0.007***	0.009***	−0.038***	−0.020***
	(0.0003)	(0.0005)	(0.001)	(0.002)	(0.0003)	(0.001)	(0.001)	(0.001)
Female	0.178***	0.197***	0.262***	0.159***	0.190***	0.226***	−0.128***	−0.172***
	(0.010)	(0.015)	(0.034)	(0.049)	(0.011)	(0.016)	(0.033)	(0.040)
Racism	0.314***	0.141***	0.225***	−0.267**	0.174***	−0.243***	0.583***	1.477***
	(0.021)	(0.034)	(0.072)	(0.116)	(0.024)	(0.038)	(0.070)	(0.095)
Political Interest	−0.210***	−0.190***	−0.253***	−0.286***	−0.052***	−0.049***	−0.347***	−0.122***
	(0.006)	(0.008)	(0.018)	(0.026)	(0.007)	(0.010)	(0.021)	(0.024)
Household Income	0.002***	0.001	0.012***	−0.012***	0.0004	0.005***	0.021***	0.020***
	(0.001)	(0.001)	(0.003)	(0.004)	(0.001)	(0.001)	(0.002)	(0.003)
Unemployed	−0.065***	0.073***	−0.210***	0.139*	−0.081***	−0.002	0.137**	−0.015
	(0.018)	(0.022)	(0.053)	(0.074)	(0.020)	(0.025)	(0.054)	(0.061)
Logged Population	0.034***	0.020***	0.076***	0.123***	0.023***	0.018***	0.043***	−0.006
	(0.003)	(0.004)	(0.010)	(0.014)	(0.003)	(0.005)	(0.010)	(0.012)
Constant			1.757***	1.958***			−1.127***	−2.370***
			(0.171)	(0.244)			(0.156)	(0.202)
Wave FEs	Y	Y	Y	Y	Y	Y	Y	Y
Observations	60,345	30,377	53,063	24,364	48,176	23,667	64,656	29,068
Log Likelihood			−12,966.410	−6,124.547			−14,621.550	−8,550.023
Akaike Inf. Crit.			25,976.810	12,293.090			29,283.090	17,140.040
Note:								*p<0.1; **p<0.05; ***p<0.01

Regression type noted above columns. Standard errors in parentheses. Two-tailed t-tests. All models use survey weights.

Table 7: Models by Party

	Dependent variable:							
	Concern		Wash Hands		Cancel Meetings		You Sick	
	ordered probit		*logistic*		*ordered probit*		*logistic*	
	D	R	D	R	D	R	D	R
	(1)	(2)	(3)	(4)	(5)	(6)	(7)	(8)
Sexism	−0.553***	−0.651***	−1.773***	−1.766***	−1.179***	−0.676***	2.858***	2.174***
	(0.043)	(0.038)	(0.153)	(0.124)	(0.047)	(0.042)	(0.115)	(0.119)
College	0.040***	0.173***	−0.027	0.350***	−0.004	0.094***	0.125***	0.189***
	(0.015)	(0.014)	(0.058)	(0.051)	(0.017)	(0.015)	(0.042)	(0.044)
White	−0.178***	−0.204***	−0.029	0.001	−0.041***	−0.162***	−0.139***	−0.130***
	(0.014)	(0.016)	(0.051)	(0.053)	(0.015)	(0.018)	(0.038)	(0.047)
Conservative (5-pt Ideology)	−0.066***	−0.233***	0.126***	−0.148***	−0.069***	−0.147***	−0.357***	−0.310***
	(0.008)	(0.007)	(0.027)	(0.022)	(0.008)	(0.007)	(0.020)	(0.017)
Age	0.011***	0.008***	0.008***	0.011***	0.010***	0.005***	−0.028***	−0.038***
	(0.0004)	(0.0004)	(0.001)	(0.001)	(0.0004)	(0.0004)	(0.001)	(0.001)
Female	0.173***	0.142***	0.125**	0.154***	0.179***	0.185***	−0.105***	−0.152***
	(0.014)	(0.012)	(0.050)	(0.042)	(0.015)	(0.014)	(0.038)	(0.040)
Racism	0.199***	0.411***	−0.319***	0.268***	−0.191***	0.283***	1.150***	0.780***
	(0.032)	(0.025)	(0.115)	(0.085)	(0.035)	(0.028)	(0.087)	(0.083)
Political Interest	−0.304***	−0.139***	−0.342***	−0.186***	−0.121***	0.006	−0.244***	−0.344***
	(0.008)	(0.008)	(0.027)	(0.024)	(0.009)	(0.009)	(0.024)	(0.027)
Household Income	0.004***	0.001	0.001	0.0005	0.005***	0.0004	0.026***	0.026***
	(0.001)	(0.001)	(0.004)	(0.003)	(0.001)	(0.001)	(0.003)	(0.003)
Unemployed	0.045**	−0.048**	−0.260***	−0.052	−0.019	−0.053**	0.133**	−0.029
	(0.022)	(0.023)	(0.073)	(0.072)	(0.023)	(0.026)	(0.060)	(0.073)
Logged Population	0.021***	0.040***	0.088***	0.096***	0.018***	0.025***	0.022**	0.015
	(0.004)	(0.004)	(0.014)	(0.012)	(0.004)	(0.004)	(0.011)	(0.012)
Constant			2.271***	2.036***			−1.732***	−0.707***
			(0.236)	(0.218)			(0.178)	(0.198)
Wave FEs	Y	Y	Y	Y	Y	Y	Y	Y
Observations	39,856	36,236	33,746	32,662	32,202	29,097	40,771	39,479
Log Likelihood			−6,523.399	−8,447.620			−10,301.510	−9,133.533
Akaike Inf. Crit.			13,088.800	16,937.240			20,641.030	18,305.060
Note:	*p<0.1; **p<0.05; ***p<0.01							

Regression type noted above columns. Standard errors in parentheses. Two-tailed t-tests. All models use survey weights.

Table 8: Models Controlling for Trump Approval

	Dependent variable:			
	Concern	Wash Hands	Cancel Meetings	You Sick
	ordered probit	*logistic*	*ordered probit*	*logistic*
	(1)	(2)	(3)	(4)
Sexism	−0.537***	−1.694***	−0.842***	2.044***
	(0.026)	(0.089)	(0.030)	(0.080)
College	0.089***	0.150***	0.028**	0.142***
	(0.010)	(0.036)	(0.011)	(0.029)
White	−0.187***	−0.048	−0.080***	−0.151***
	(0.010)	(0.033)	(0.011)	(0.028)
Democrat	0.227***	0.346***	0.135***	0.291***
	(0.013)	(0.043)	(0.015)	(0.043)
Republican	0.013	0.267***	−0.040***	−0.083*
	(0.014)	(0.043)	(0.016)	(0.044)
Conservative (5-pt Ideology)	−0.141***	−0.026*	−0.116***	−0.320***
	(0.005)	(0.015)	(0.005)	(0.012)
Age	0.009***	0.007***	0.007***	−0.030***
	(0.0003)	(0.001)	(0.0003)	(0.001)
Female	0.167***	0.192***	0.188***	−0.104***
	(0.008)	(0.029)	(0.009)	(0.026)
Racism	0.328***	0.204***	0.100***	0.643***
	(0.018)	(0.063)	(0.020)	(0.057)
Political Interest	−0.206***	−0.284***	−0.061***	−0.227***
	(0.005)	(0.016)	(0.006)	(0.017)
Household Income	0.002**	0.002	0.002***	0.022***
	(0.001)	(0.002)	(0.001)	(0.002)
Unemployed	−0.020	−0.105**	−0.055***	0.088**
	(0.014)	(0.045)	(0.016)	(0.043)
Logged Population	0.028***	0.091***	0.019***	0.032***
	(0.003)	(0.009)	(0.003)	(0.008)
Pres Approval	−0.120***	−0.155***	−0.124***	0.377***
	(0.005)	(0.016)	(0.005)	(0.014)
Constant		2.248***		−2.347***
		(0.153)		(0.135)
Wave FEs	Y	Y	Y	Y
Observations	86,679	74,560	69,616	90,290
Log Likelihood		−17,883.930		−21,690.700
Akaike Inf. Crit.		35,815.860		43,425.390
Note:				*p<0.1; **p<0.05; ***p<0.01

Regression type noted above columns. Standard errors in parentheses. Two-tailed t-tests. All models use survey weights.

References

Bracic, A., M. Israel-Trummel, and A. F. Shortle (2019). Is Sexism for White People? Gender Stereotypes, Race, and the 2016 Presidential Election. *Political Behavior 41*(2), 281–307.

Cassese, E. C. and T. D. Barnes (2019). Reconciling Sexism and Women's Support for Republican Candidates: A Look at Gender, Class, and Whiteness in the 2012 and 2016 Presidential Races. *Political Behavior 41*(3), 677–700.

Cassese, E. C. and M. R. Holman (2019). Playing the Woman Card: Ambivalent Sexism in the 2016 U. S. Presidential Race. *Political Psychology 40*(1), 55–74.

Frasure-Yokley, L. (2018). Choosing the Velvet Glove: Women Voters, Ambivalent Sexism, and Vote Choice in 2016. *Journal of Race, Ethnicity, and Politics 3*, 3–25.

McDermott, M. (2016). *Masculinity, Femininity, and American Political Behavior*. New York, NY: Oxford University Press.

Schaffner, B., M. MacWilliams, and T. Nteta (2018). Understanding White Polarization in the 2016 Vote for President: The Sobering Role of Racism and Sexism. *Political Science Quarterly 133*(1), 9–34.

Schaffner, B. F. (2020). The Heightened Importance of Racism and Seixsm in the 2018 U.S. Midterm Elections. *British Journal of Political Science Forthcomin*.

Sides, J., L. Vavreck, and M. Tesler (2018). *Identity Crisis: The 2016 Presidential Campaign and the Battle for the Meaning of America*. Princeton, NJ: Princeton University Press.

Smiler, A. P. (2006). Conforming to Masculine Norms: Evidence for Validity among Adult Men and Women. *Sex Roles2 54*(11), 767–775.

Swim, J. K., K. J. Aikin, W. S. Hall, and B. A. Hunter (1995). Sexism and Racism : Old-Fashioned and Modern Prejudices. *Journal of Personality and Social Psychology 68*(2), 199–214.

Tausanovitch, C. and L. Vavreck (2020). Democracy Fund + UCLA Nationscape.

Tausanovitch, C., L. Vavreck, T. Reny, A. R. Hayes, and A. Rudkin (2019). Democracy Fund + UCLA Nationscape Methodology and Representativeness Assessment.

Valentino, N. A., C. Wayne, and M. Oceno (2018). Mobilizing Sexism: The Interaction of Emotion and Gender Attitudes in the 2016 U.S. Presidential Election. *Public Opinion Quarterly 82*, 799–821.
